# The Danger of Administering Chloroform in the Presence of an Open Flame

**Published:** 1891-07

**Authors:** 


THE DANGER OF ADMINISTERING CHLOROFORM IN THE PRESENCE OF AN OPEN FLAME.—Phosgene (*Med. Record*), a gas exceedingly irritating to the respiratory tract, is produced by the decomposition of chloroform in the presence of an open flame. While no cases are recorded in which death could be directly traceable to this source, the question suggests itself whether it may not have been the exciting cause of fatal broncho-pneumonia in some cases after operation.—*Memphis Jour. Med. Sciences.*

